# Event and Comment

**Published:** 1915-09

**Authors:** 


### DENTAL REGISTER

Vol. LXIX SEPTEMBER, 1915 No. 9

### EVENT AND COMMENT

THE FUTURE OF PREVENTIVE DENTISTRY. As we look back over the history of dentistry as a healing art, we realize that dentistry is entitled to the classification of a beneficent calling, because of the great good it has been able to do to suffering humanity. We also feel justified in claiming it to be an important branch or member of the great art and profession of medicine. In a very peculiar sense dentistry has many claims to even a separate and distinct professional classification. It has not only developed its art to a degree of perfection that has given it world-wide recognition; but it has gone farther, and to a large extent mastered the fundamental sciences upon which its art is founded. Like every other useful art its technical practices have to a large extent preceded and made necessary the scientific explanations for its practices. We have many reasons to be proud of the marvelous advancement made by our profession in the more recent years.

After all that can be said, however, is it to be along these same lines of progress that the greatest good is to come to humanity? There is a general expectancy in the minds of the people that the day of disease is fast going by and that the new day will bring much relief from suffering. We may see evidences of this in the popular stampede to various forms of mental healing that are so popular, and to the great activities for public hygiene propaganda in almost every direction. It will not do for us to overlook these manifestations of the coming change as a popular stampede of the unlearned, for the reason that the educated laymen and medical men are strongly supporting these movements. Even our best medical scientists are devoting their greatest energies to the discovery of prophylactic agencies. Our own profession is no exception. Some of the most capable and serious-minded men and women in our profession are devoting much of their strength and energies toward preventive dentistry. It is easy for one who has kept his eyes open to what was going on in dental practice for the past eight or ten years, to see the trend of thought and activities that has brought us squarely up to the fact that dentistry of the future, to be classified professionally, must mean something else than mere rescue procedures. In fact, it would seem that public opinion is already demanding why we are not doing more to stay the progress of the most prevalent disease known to humanity. Perhaps we are doing all that we know how to do, by our constant efforts to teach people the value of their teeth and the necessity for absolute oral cleanliness. If this, however, has been found lacking or impracticable, is there not some other solution that we may hope to develop by persistent effort and scientific research? We do not, of course, wish to be understood as deprecating the splendid achievements of dental technicians or scientists, or to infer that the oral hygiene propaganda has failed to accomplish that which its promoters expected and promised. The public propaganda for good teeth has done a lot of commendable work. It has developed an enormous demand for dentrifices which is being adequately supplied. The school clinics have prospered and made demands for large facilities for caring for the teeth of poor children; and millions of dollars from great philanthropists and municipalities are being diverted to this grand work in oral hygiene. All of this is of tremendous importance and we rejoice exceedingly in realizing that it is a great step in advance of anything the world has heretofore known. Shall we rest satisfied with this pleasing outlook, or can we see a still more hopeful future, when our highest ideals of absolute freedom from dental disease may become a realized fact?

To our mind it is a practicable possibility by present known methods of oral hygiene to secure practically normal dentition and to keep the teeth from seriously impaired function during the active period of human existence. If this is possible, it certainly is ideal and worth while striving for. We haven't space here to develop the process, but it will, perhaps, be sufficient to say that it means an earlier and somewhat modified method of dealing with the problem of oral hygiene with children. There is no adequate reason why the first teeth of the child should be malposed or suffer from caries, or that there should be a maldevelopment of their jaws or facial bones. If the oral hygienist wants to prevent dental disease he must begin further back in the child's life than is now believed necessary. The prenatal period must have careful consideration from the medical man and the dentist should be put in consultation with the medical man as soon as the first teeth are ready to erupt and he should have the child's mouth under surveillance until the permanent denture is fully erupted, and at no time in this period should a malformation or injury of any kind to a single tooth be permitted. A service of this kind is practicable, and if scientifically carried out, will result in a perfectly developed dental organism, which can be kept free from disease, and which incidentally and necessarily will result in a more perfect physical development than we have known and a superhuman body that will be capable of resisting disease of all kinds and that will be equal to the demands made by modern civilization on mankind.

DEFENDING THE PROFESSION FROM MALPRACTICE SUITS. The Law Committee of the National Dental Association, is proposing to defend the members of that Association from malpractice suits, and thinks it feasible to do so at an average expense of twenty-five cents per member each year. This is certainly cheap enough. The fact is that malpractice suits against good practitioners are rather rare Such suits are more common in the practice of medicine. The dental profession seems to have suffered more from the patent sharks than from any other cause. This fact makes the suggestion pertinent that it might be a good plan for the National Dental Association to take over the Dental Protective Association and other similar organizations, and by employing a good lawyer the protection of its members from unjust law suits in this direction also could be taken care of. With the funds already on hand in these organizations and a part of the annual dues of its members an endowment fund would be created in a few years which would be ample to take care of all such litigation. There are difficulties in the way, but none that are insurmountable. Such a scheme would bind the profession closer together and would make a membership in the National organization a desirable thing for every practitioner.

